# Patient Satisfaction with and Use of Telemental Health Services in the Perinatal Period: a Survey Study

**DOI:** 10.1007/s11126-020-09874-8

**Published:** 2021-01-03

**Authors:** Marra Ackerman, Elizabeth Greenwald, Paraskevi Noulas, Christina Ahn

**Affiliations:** 1 Department of Psychiatry, NYU Grossman School of Medicine, New York, NY USA; 2 Department of Psychiatry, Beth Israel Deaconess Medical Center, Boston, MA USA

**Keywords:** Perinatal, Postpartum, Telemental health, Depression, Anxiety

## Abstract

We aimed to assess patients’ utilization of and satisfaction with telemental health (TMH) in the perinatal period. We hypothesized that satisfaction with TMH would be at least equal to, if not greater than, with in-person appointments. We conducted a cross-sectional survey between March 2018–June 2019 to evaluate patient satisfaction with and use of TMH services in the perinatal period. Participants used TMH services across the second and third trimester of pregnancy and the first year post-partum. Nearly half of the patients (8/19, 42%) used TMH to see their provider within the first two weeks post-partum. Participants were most commonly in treatment for anxiety (14/19, 74%) and/or depression (9/19, 47%). Most participants agreed or strongly agreed (13/19, 69%) that TMH improved their access to healthcare and that they could see the clinician as well as if they met in person (14/19, 74%). TMH was a highly accepted and appreciated method of mental health care delivery for perinatal women when offered as an alternative to in-person or telephone sessions.

**Supplementary Information:**

The online version contains supplementary material available at 10.1007/s11126-020-09874-8.

## Background

Prior to March 2020, telemental health (TMH) was minimally utilized given technological, legal and regulatory concerns [1]. Since the start of the COVID-19 pandemic, mental health treatment has been largely provided via TMH. By in large, providers and patients have quickly learned to adjust to TMH [2]. Pre-pandemic, TMH improved patients’ access to care by removing transportation barriers, reducing missed work hours, and relieving caregiver burden of accompanying patients to appointments [3]. Evidence indicates TMH can improve outcomes including fewer hospital readmissions, better adherence, and shorter hospital stays [3, 4]. There have been limited available studies of TMH in the perinatal period. In 2017, Wisner et al. showed that telephone-delivered care to women with depression in the post-partum period had comparable positive impact on symptoms and functioning as compared to usual care [5].

More research is needed to explore potential benefits of TMH in the perinatal population [6]. The perinatal period, defined as the 40 week gestational period and first year post-partum, is associated with many stressors including: role transition to motherhood; relationship stressors; schedule changes; and decreased sleep. Physical changes (such as hormonal fluctuations and recovery from labor) also affect patients’ mobility and ability to access care. Psychotropic medication regimens may also be changed due to concerns for risk to the fetus or during lactation. The point prevalence of depression during pregnancy is estimated between 11 and 15% and 13–19% in the post-partum [7]. The US Preventative Services Task Force recommends screening for depression in pregnant and postpartum women and referring high risk women to counseling interventions [8].

Due to the many challenges associated with the perinatal period, TMH may prove particularly valuable in this population, beyond the current pandemic [9]. TMH can improve patient access to reproductive psychiatrists who may be concentrated in academic medical centers; reduce transportation costs and childcare burdens; and allow for clinical observation of the patient and her baby in their home environment, allowing for a direct assessment of bonding and attachment. As such, in this present study, we aimed to assess patients’ utilization of and satisfaction with TMH in the perinatal period. We hypothesized that satisfaction with TMH would be at least equal to, if not greater than, with in-person appointments.

## Methods

We conducted a cross-sectional survey between March 2018–June 2019 to evaluate patient satisfaction with and use of TMH services in the perinatal period. Participants were eligible for inclusion if they were female; between the ages of 18–50 years; English speaking; and had utilized TMH health services at NYU Psychiatry Associates during pregnancy and/or the first year post-partum within a year of recruitment. Patients were able to connect with their providers through any device that had a microphone and camera (e.g. smartphone, tablet or desktop) and were able to access their sessions from work or home so long as they were in a private, quiet, and safe space. TMH sessions were conducted via an encrypted HIPAA-compliant platform through the NYULH MyChart portal. Patients were provided with tip sheets assisting them with the login process and help desk contact information for any technical questions or issues. Eligible patients who were informed of the study and agreed to be contacted were invited to complete an online de-identified survey via email using REDCap. The survey required approximately 5–10 min to complete.

The survey began with 18 fields regarding the patient’s demographic information, recent pregnancy, and experience using telehealth services. It then included 21 questions adapted from the Telehealth Usability Questionnaire [10], which assessed the usefulness of TMH in the perinatal period; ease of use and learnability; interface quality and reliability; satisfaction with TMH, and plans for future use. Two questions were added that were specific to the post-partum period (see Table 1: questions 22–23). Patients were also asked whether or not they planned to continue to use TMH, and space was provided for additional comments on TMH.

**Table 1:** Telehealth Usability Questionnaire Results

	N/A	Strongly disagree	Disagree	More or less disagree	Neither agree nor disagree	More or less agree	Agree	Strongly agree
(1) Telehealth improves my access to healthcare services.	0	1 (5.3)	0	0	4 (21.1)	1 (5.3)	3 (15.8)	**10 (52.6)**
(2) Telehealth saves me time traveling to a hospital or specialist clinic.	0	1 (5.3)	0	0	0	0	1 (5.3)	**17 (89.5)**
(3) Telehealth provides for my healthcare need.	0	1 (5.3)	1 (5.3)	0	0	0	6 (31.6)	**11 (57.9)**
(4) It was simple to use this system.	0	1 (5.3)	0	0	0	2 (10.5)	5 (26.3)	**11 (57.9)**
(5) It was easy to learn to use the system.	0	1 (5.3)	0	0	0	1 (5.3)	5 (26.3)	**12 (63.2)**
(6) I believe I could become productive quickly using this system.	0	1 (5.3)	0	1 (5.3)	0	0	6 (31.6)	**11 (57.9)**
(7) The way I interact with this system is pleasant.	0	0	2 (10.5)	0	0	4 (21.1)	4 (21.1)	**9 (47.4)**
(8) I like using the system.	1 (5.3)	1 (5.3)	0	0	2 (10.5)	1 (5.3)	4 (21.1)	**10 (52.6)**
(9) The system is simple and easy to understand.	0	1 (5.3)	0	0	0	2 (10.5)	6 (31.6)	**10 (52.6)**
(10) This system is able to do everything I would want it to be able to do.	0	1 (5.3)	2 (10.5)	1 (5.3)	0	2 (10.5)	5 (26.3)	**8 (42.1)**
(11) I can easily talk to the clinician using the telehealth system.	0	2 (10.5)	0	2 (10.5)	0	1 (5.3)	4 (21.1)	**10 (52.6)**
(12) I can hear the clinician clearly using the telehealth system.	0	1 (5.3)	2 (10.5)	0	2 (10.5)	1 (5.3)	4 (21.1)	**9 (47.4)**
(13) I felt I was able to express myself effectively.	0	1 (5.3)	0	0	4 (21.1)	0	5 (26.3)	**9 (47.4)**
(14) Using the telehealth system, I can see the clinician as well as if we met in person.	0	3 (15.8)	1 (5.3)	0	0	1 (5.3)	5 (26.3)	**9 (47.4)**
(15) I think the visits provided over the telehealth system are the same as in-person visits.	1 (5.3)	2 (10.5)	1 (5.3)	0	0	4 (21.1)	3 (15.8)	**8 (42.1)**
(16) Whenever I made a mistake using the system, I could recover easily and quickly.	3 (15.8)	1 (5.3)	0	0	1 (5.3)	2 (10.5)	4 (21.1)	**8 (42.1)**
(17) The system gave error messages that clearly told me how to fix problems.	**6 (31.6)**	2 (10.5)	2 (10.5)	2 (10.5)	2 (10.5)	1 (5.3)	3 (15.8)	1 (5.3)
(18) I feel comfortable communicating with the clinician using the telehealth system.	1 (5.3)	1 (5.3)	1 (5.3)	0	0	1 (5.3)	6 (31.6)	**9 (47.4)**
(19) Telehealth is an acceptable way to receive healthcare services.	0	1 (5.3)	1 (5.3)	0	1 (5.3)	1 (5.3)	4 (21.1)	**11 (57.9)**
(20) I would use telehealth services again.	0	1 (5.6)	1 (5.6)	0	0	0	4 (22.2)	**12 (66.7)**
(21) Overall, I am satisfied with this telehealth system.	0	2 (10.5)	0	0	1 (5.3)	1 (5.3)	7 (36.8)	**8 (42.1)**
*(22) Telehealth services allowed me to see my provider sooner in post-partum period than if only in-person visits were available.	5 (26.3)	0	1 (5.3)	0	0	0	3 (15.8)	**10 (52.6)**
*(23) It was valuable for the provider to see me with my baby at home rather than in the office.	**7 (36.8)**	0	0	0	3 (15.8)	2 (10.5)	3 (15.8)	4 (21.1)

*Questions 22–23 were added to the Telehealth Usability Questionnaire [10]

Survey responses were linked to a de-identified REDCap ID number and were maintained in an encrypted file by a study staff member who was not directly involved in patient care. Descriptive and correlational statistics were used for data analysis.

## Results

From March 2018–June 2019, 45 perinatal patients at NYU Psychiatry Associates were offered TMH; 5 were ineligible due to living out of state, and 8 declined; 32 were surveyed, and 19 responded to the online survey (59% response rate). Enrollment ended in June 2019. Sociodemographic information is depicted in Table 2. Most patients were between the ages of 31–35 (13/19, 68.4%), married (18/19, 94.7%), employed full-time (17/19, 89.5%), and Caucasian (16/19, 84.2%). The majority of patients were pregnant or had one child (16/19, 84.2%). All had at least a college degree. Over half of the patients’ reported household income greater than $200,000 (11/19, 57.9%), and no patients reported household income less than $50,000 per year. More than half of the patients (11/19, 57.9%) would have needed to commute more than 45 min for an in-office appointment. Most participants used TMH for a combination of medication management and psychotherapy (12/19, 63%). The large majority of patients had the same insurance coverage for TMH as for in-person visits (14/19, 74%).

**Table 2:** Demographic Characteristics

Characteristic		All Patients (*N* = 19)	%
Weeks Pregnant	N/A (post-partum)	14	73.7
	2nd trimester (13–27 weeks)	3	15.8
	3rd trimester (28–41 weeks)	2	10.5
Months post-partum	N/A (still pregnant)	5	26.3
	1 month or less	3	15.8
	2–6 months	5	26.3
	7–12 months	4	21.1
	More than 12 months	2	10.5
Age	26–30	3	15.8
	31–35	13	68.4
	36–40	3	15.8
Current marital status	Married (or in a domestic partnership)	18	94.7
	Divorced	1	5.3
Current employment status	Full-time employed	17	89.4
	Homemaker	1	5.3
	Unable to work due to disability	1	5.3
Highest degree of education	College degree	8	42.1
	Graduate degree	11	57.9
Race/ethnicity	Caucasian/White	16	84.2
	Black or African-American	1	5.3
	Asian/Pacific Islander	2	10.5
Annual household income	50–100,000 per year	4	21.1
	101,000–150,000 per year	4	21.1
	> 200,000 per year	11	57.8
Distance from NYU Psychiatry Associates 1 Park Ave office	< 5 miles	7	36.8
	5–10 miles	2	10.5
	11–20 miles	6	31.6
	21–30 miles	1	5.3
	> 30 miles	2	10.5
	I don’t know	1	5.3
Time to travel to NYU Psychiatry Associates 1 Park Ave office	16–30 minutes	6	31.6
	31–45 minutes	2	10.5
	46–60 minutes	7	36.8
	> 60 minutes	4	21.1
Number of children	0	5	26.3
	1	11	57.9
	2	3	15.8
Mode of delivery for most recent pregnancy	N/A (still pregnant)	5	26.3
	Vaginal delivery	9	47.4
	C-section	5	26.3
Method of feeding for youngest child during the first six months	N/A (still pregnant)	5	26.3
	Exclusive breastfeeding	4	21.1
	Exclusive bottlefeeding	4	21.1
	Combination of above	6	31.5
Total number of telehealth visits during pregnancy and the first year post-partum	1–3	12	63.2
	4–6	2	10.5
	7–10	3	15.8
	> 10	2	10.5
Type of treatment for which telehealth services were used	Medication management	4	21.0
	Psychotherapy	3	15.8
	Combination of above	12	63.2
Insurance coverage for telehealth services	Same coverage for telehealth as for in-person visits	14	73.7
	No coverage for telehealth or in-person visits	2	10.5
	I don’t know	3	15.8
Planning to continue to use telehealth services	Yes	16	84.2
	No	3	15.8

Participants used TMH services across the second and third trimester of pregnancy and the first year post-partum. Notably, nearly half of the patients (8/19, 42%) used TMH to see their provider within the first two weeks post-partum. Participants were most commonly in treatment for anxiety (14/19, 74%) and/or depression (9/19, 47%) (Supplementary Table 1). As seen in Table 1, most participants agreed or strongly agreed (13/19, 69%) that TMH improved their access to healthcare and that they could see the clinician as well as if they met in person (14/19, 74%). Comments from patients included: “[TMH was] incredibly convenient and necessary for post-partum period. I would not have been able to access care without the teleservice option. The audio was unclear at times, but manageable” and “The telehealth system is very convenient for me to use because I cannot commute with my baby... It makes seeing my doctor of choice a pleasant experience when and where it is best for me.”

## Conclusions

Our survey results indicate that TMH in the perinatal period improves access to healthcare; is easy to use and to learn; and provides a satisfying means of accessing mental healthcare. Some clinicians have expressed concern that TMH creates a metaphorical distance between the provider and patient, which may negatively affect patient satisfaction and outcomes. However, evidence from multiple studies, including the present study, shows that patients are largely as satisfied with TMH as they are with in-person visits [3, 11]. Randomized controlled trials have also shown equal outcomes for patients utilizing TMH as compared to in-person visits [4, 12].

Many TMH interventions have understandably targeted rural areas to improve access to care. This study highlights the utility of TMH in an urban environment where physical distance may not be an obvious obstacle, but commuting time and travel with an infant can pose significant barriers. TMH offers the opportunity to see women earlier in the post-partum period than is often possible with in-office visits. TMH thereby can provide an opportunity for earlier intervention and for observation of the mother-child dyad in the home environment. The most notable barrier to receiving TMH services was licensing issues which preventing clinicians from providing out of state services. Secondary obstacles were infrequent technical issues and not having TMH set up prior to the immediate postpartum period.

Prior to the COVID-19 pandemic, licensure restriction prevented clinicians from seeing out-of-state patients. Emergency waivers of licensure requirements during the pandemic have helped support treatment continuity and expand access to care. Healthcare providers have been lobbying for an overhaul of the state licensure restrictions as maintaining multiple state licenses is both arduous and expensive.

This study is limited by the relatively small population size and the homogenous nature of the sample as a wealthy, well-educated, Caucasian cohort seen in an academic group practice. However, it is worth noting that TMH services delivered in this study were largely compensated by insurance at the same rate as in-person visits; saved the cost of commuting; and required only a smartphone, tablet, or desktop with access to the internet. Future studies should examine the accessibility of TMH for perinatal women in other sociodemographic and ethnic groups.

In sum, this study illustrates that even prior to the COVID-19 pandemic, TMH was a highly accepted and appreciated method of mental health care delivery for perinatal women when offered as an alternative to in-person or telephone sessions. TMH offers opportunities for earlier intervention and expansion of access to specialized reproductive psychiatric care. The benefits of telemedicine delivered across state lines during the pandemic will hopefully set a precedent that will continue even after emergency clauses are lifted.

## Supplementary Information

ESM 1 (DOCX 14 kb)

## Data Availability

Data has been collected and maintained as de-identified data in REDcap.

## References

[1] Abrams J, Sossong S, Schwamm LH, Barsanti L, Carter M, Kling N, Kotarski M, Leddy J, Meller B, Simoni M, Sullivan M, Wozniak J (2017). Practical issues in delivery of clinician-to-patient Telemental health in an Academic Medical Center. Harv Rev Psychiatry.

[2] Chen JA, Chung WJ, Young SK, Tuttle MC, Collins MB et al (2020) COVID-19 and telepsychiatry: early outpatient experiences and implications for the future. Gen Hosp Psychiatry 66:89–95. Available from: 10.1016/j.genhosppsych.2020.07.002. 10.1016/j.genhosppsych.2020.07.002 PMC7347331 32750604

[3] Waugh M, Voyles D, Thomas MR (2015). Telepsychiatry: benefits and costs in a changing health-care environment. Int Rev Psychiatry.

[4] Hilty DM, Ferrer DC, Parish MB, Johnston B, Callahan EJ, Yellowlees PM (2013). The effectiveness of telemental health: a 2013 review. Telemed J E Health.

[5] Wisner KL, Sit DKY, McShea M, Luther JF, Eng HF, Dills JL, Moses-Kolko EL, Wisniewski SR (2017). Telephone-based depression Care Management for Postpartum Women: a randomized controlled trial. J Clin Psychiatry Nov/Dec.

[6] Nair U, Armfield NR, Chatfield MD, Edirippulige S (2018) The effectiveness of telemedicine interventions to address maternal depression: a systematic review and meta-analysis. J Telemed Telecare 24(10):639–650. Available from: 10.1177/1357633X18794332. 10.1177/1357633X18794332 30343660

[7] Howard LM, Molyneaux E, Dennis CL, Rochat T, Stein A et al (2014) Non-psychotic mental disorders in the perinatal period. The Lancet 384(9956):1775-1788. Available from: 10.1016/S0140-6736(14)61276-9. 10.1016/S0140-6736(14)61276-9 25455248

[8] US Preventive Services Task Force (2019) Interventions to Prevent Perinatal Depression: US Preventive Services Task Force Recommendation Statement. JAMA 321(6):580–587. Available from: doi:10.1001/jama.2019.0007 10.1001/jama.2019.0007 30747971

[9] van den Heuvel JF, Groenhof TK, Veerbeek JH, van Solinge WW, Lely AT et al (2018) eHealth as the next-generation perinatal care: an overview of the literature. J Med Internet Res Jun 5;20(6):e202. Available from: doi: 10.2196/jmir.9262. 10.2196/jmir.9262 PMC6008510 29871855

[10] Parmanto B, Lewis AN, Graham KM, Bertolet MH (2016). Development of the Telehealth usability questionnaire (TUQ). Int J Telerehabil.

[11] Malhotra S, Chakrabarti S, Shah R (2013) Telepsychiatry: promise, potential, and challenges. Indian J psychiatry 55(1):3–11. Available from: 10.4103/0019-5545.105499. 10.4103/0019-5545.105499 PMC3574452 23441027

[12] García-Lizana F, Muñoz-Mayorga I (2010) What about telepsychiatry? A systematic review. Prim Care Companion J Clin Psychiatry 12(2):PCC.09m00831. Available from: 10.4088/PCC.09m00831whi. 10.4088/PCC.09m00831whi PMC2911004 20694116

