# Supplementary material for: Patient Satisfaction with and Use of Telemental Health Services in the Perinatal Period: a Survey Study
Source: Psychiatr Q. 2021 Jan 3;92(3):925–33. doi: 10.1007/s11126-020-09874-8 (PMC7778562; doi:10.1007/s11126-020-09874-8)
Supplement: Supplementary file 1 — (DOCX 14 kb) [file 11126_2020_9874_MOESM1_ESM.docx]

**Supplementary Table 1**

| Characteristic |  | All Patients (N = 19); 1 or more answers per participant |
| --- | --- | --- |
| Reason for seeing a provider at NYU Psychiatry Associates (1 or more answers per participant) | ADHD  Anxiety disorder  Bipolar disorder  Depression  Obsessive-compulsive disorder  Psychosis | 1  14  1  9  2  1 |
| Stage of pregnancy and/or post-partum during which telehealth services were used (1 or more answers per participant) | First trimester (Less than or equal to 12 weeks)  Second trimester (13-27 weeks)  Third trimester (28-41 weeks)  0-2 weeks post-partum  3-12 weeks post-partum  4-6 months post-partum  7-12 months post-partum  More than 12 months post-partum | 4  7  5  8  8  4  4  1 |
